# Use of Nanotechnology to Develop Multi-Drug Inhibitors For Cancer Therapy

**DOI:** 10.4172/2157-7439.1000184

**Published:** 2013-12

**Authors:** Raghavendra Gowda, Nathan R. Jones, Shubhadeep Banerjee, Gavin P. Robertson

**Affiliations:** 1 Department of Pharmacology, The Pennsylvania State University College of Medicine, Hershey, PA 17033, USA; 2 Department of Pathology, The Pennsylvania State University College of Medicine, Hershey, PA 17033, USA; 3 Department of Dermatology, The Pennsylvania State University College of Medicine, Hershey, PA 17033, USA; 4 Department of Surgery, The Pennsylvania State University College of Medicine, Hershey, PA 17033, USA; 5 Penn State Hershey Melanoma Center, The Pennsylvania State University College of Medicine, Hershey, PA 17033, USA; 6 Penn State Melanoma Therapeutics Program, The Pennsylvania State University College of Medicine, Hershey, PA 17033, USA; 7 The Foreman Foundation for Melanoma Research, The Pennsylvania State University College of Medicine, Hershey, PA 17033, USA

**Keywords:** Nanotechnology, Multi-target inhibitors, Multi-drug inhibitors, Nanoliposomes, Cancer therapy

## Abstract

Therapeutic agents that inhibit a single target often cannot combat a multifactorial disease such as cancer. Thus, multi-target inhibitors (MTIs) are needed to circumvent complications such as the development of resistance. There are two predominant types of MTIs, (a) single drug inhibitor (SDIs) that affect multiple pathways simultaneously, and (b) combinatorial agents or multi-drug inhibitors (MDIs) that inhibit multiple pathways. Single agent multi-target kinase inhibitors are amongst the most prominent class of compounds belonging to the former, whereas the latter includes many different classes of combinatorial agents that have been used to achieve synergistic efficacy against cancer. Safe delivery and accumulation at the tumor site is of paramount importance for MTIs because inhibition of multiple key signaling pathways has the potential to lead to systemic toxicity. For this reason, the development of drug delivery mechanisms using nanotechnology is preferable in order to ensure that the MDIs accumulate in the tumor vasculature, thereby increasing efficacy and minimizing off-target and systemic side effects. This review will discuss how nanotechnology can be used for the development of MTIs for cancer therapy and also it concludes with a discussion of the future of nanoparticle-based MTIs as well as the continuing obstacles being faced during the development of these unique agents.’

## What are Multi-Target Inhibitors (MTIs), and why are they Needed?

During the past several decades, there has been significant progress in treating cancer with chemotherapy, radiation, and surgery [1]. However, traditional methods of cancer treatment are frequently limited because of systemic side effects, the development of resistance, and sub-optimal drug concentrations at the tumor site [2, 3]. Many recent advances in cancer therapy have centered on targeting oncogenes involved in proliferation and survival pathways specific to cancer cells [4–6]. Several targeted therapies that have enjoyed great success are Zelboraf (vemurafenib; formerly known as PLX4032) for treating advanced melanoma [7], monoclonal antibody trastuzumab for human epidermal growth factor receptor 2 (HER2) positive breast cancer [8], imatinib for break cluster region-abelson leukemia (BCR-Abl) positive chronic myelogenous leukemia (CML) and gastrointestinal stromal tumors (GIST) [9,10], and gefitinib and erlotinib for non-small cell lung cancer (NSCLC) [11]. However, in many cases, cancer patients develop resistance when treated with therapies that target single pathways because the multi-genic abnormalities present in cancer cells allow them to circumvent the action of these agents. The ability of advanced melanoma to develop resistance to Zelboraf is a recent example of how tumors can bypass the point of inhibition, leading to disease recurrence and progression [12,13]. Because it is often true that single-target agents cannot combat a multifactorial disease such as cancer, multi-target inhibitors (MTIs) are becoming more and more attractive in cancer therapy as they are often more effective and less prone to resistance development than monotherapies.

### Single agent Multi-Target Inhibitors (MTIs)

**Figure 1:**
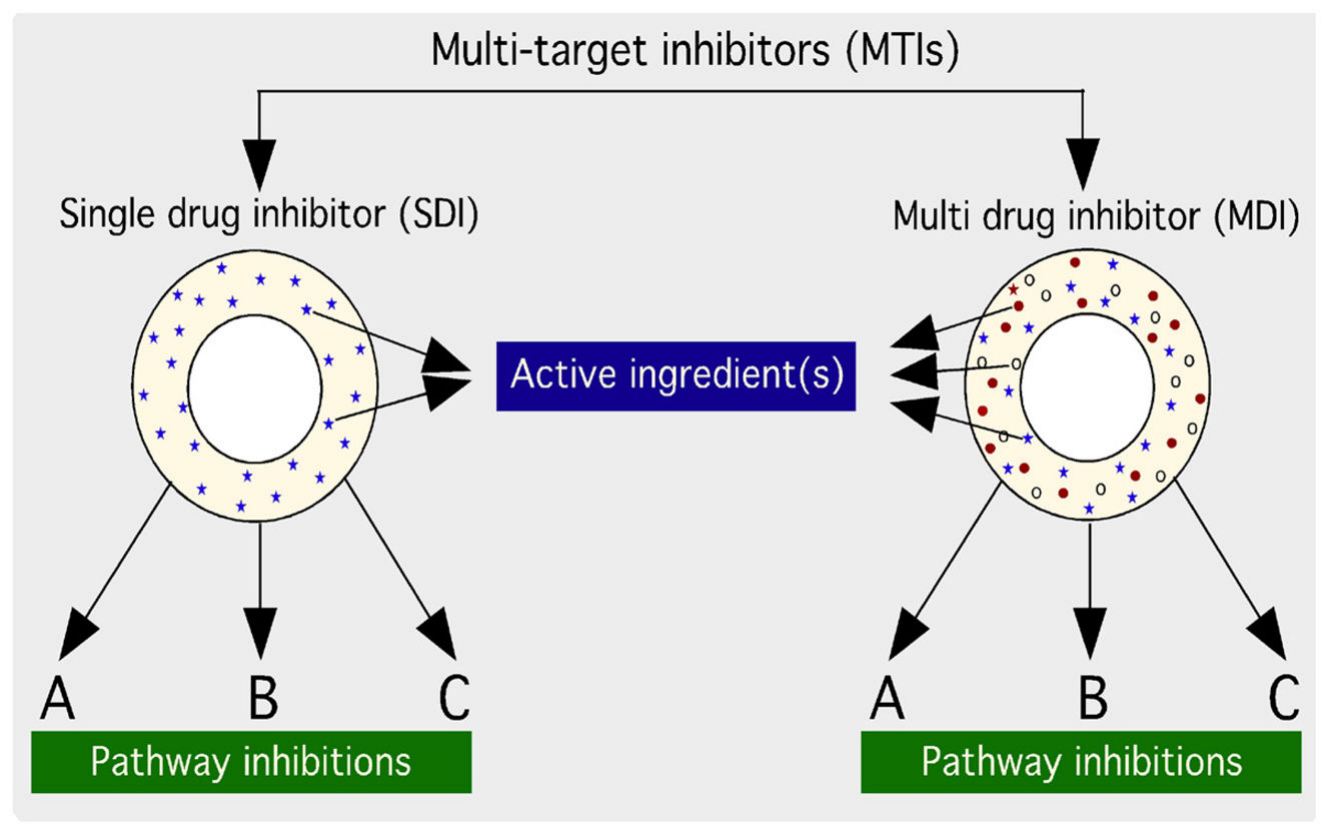
Schematic of multi-target inhibitors (MTI) that affect multiple pathways simultaneously to inhibit cancer cell growth and survival.

MTIs can be either single drugs that inhibit multiple targets (SDIs), or a combination of multiple agents “Multi-Drug inhibitors” (MDIs) that synergistically inhibit multiple pathways (Figure 1). The most common types of single-drug MTIs are small molecule kinase inhibitors, which are continually being evaluated as new anticancer therapies (Table 1). Small molecule kinase inhibitors are used most commonly as MTIs because deregulation of kinase activity is a major mechanism by which cancer cells evade normal controls regulating cell proliferation and survival [14]. There are roughly 500 kinases in the human kinome, and there are often multiple aberrant kinase pathways involved in a single tumor. Kinase inhibitors are often designed to target oncogenic receptors such as vascular endothelial growth factor receptor (VEGFR), endothelial growth factor receptor (EGFR), platelet-derived growth factor receptor (PDGFR), or they may be designed to inhibit downstream intracellular kinases or pathways such as tyrosine-protein kinase (cSrc) and mitogen activated protein (MAPK) pathways.

It is also increasingly apparent that simultaneous inhibition of factors within the tumor microenvironment is necessary and MTIs might be helpful for this application. Hypoxic tumor microenvironment forces tumors to acquire additional vascularization, which requires aberrant vascular endothelial growth factor (VEGF) signaling [15]. Carcinoma cells can recruit macrophages, lymphocytes, and mast cells and form an aberrant paracrine loop, which causes these cells to secrete VEGF and other factors that provide the cancer cells with additional nutrients and vascularization [16]. This additional leaky vasculature also provides an escape route for metastatic cells, which is further exacerbated by macrophage secretion of matrix metalloproteinases (MMPs) [17]. Furthermore, paracrine signaling by carcinoma cells can also lead to fibroblast activation and a subsequent change in the production of growth factors such as platelet-derived growth factor (PDGF) and transforming growth factor-β (TGF-β) [18].

Fibroblasts can also impact disease progression by secreting MMPs that act on the extracellular matrix (ECM) surrounding the tumor [19]. These types of paracrine loops are well established in many cancers [20–25], and thus the future of cancer therapy relies on targeting pathways present within the tumor cells as well as within the cells of the tumor microenvironment. This method has the potential to be particularly effective, because the microenvironment includes normal stromal cells that do not have the same proclivities towards resistance, which means MTIs affecting neoplastic cells and the microenvironment may further aid in the prevention of resistant disease [26].

The use of rational drug design to inhibit specific kinases in different cancer types has vastly improved cancer therapy [27]. There are a number of examples of multi-target kinase inhibitors currently on the market that inhibit multiple oncogenic pathways within tumors simultaneously, including sorafenib (inhibits MAPK, VEGFR, PDGFR, and mast/stem cell growth factor receptor (cKit) for the treatment of renal cell carcinoma (RCC) and hepatocellular carcinoma (HCC) [28,29], vandetanib (inhibits rearranged during transfection (RET) kinase, VEGFR, and EGFR) for treatment of thyroid tumors [30], and pozopanib (inhibits PDGFR, VEGFR, and cKit) for treatment of RCC and sarcoma [31,32]. When designing these types of agents, it is necessary to understand the structural basis of individual kinases in order to achieve selective inhibition. While there has been success in rational drug design for cancer therapy, only a few pathways are druggable with current chemistries [33,34]. This means that few first-in-class inhibitors enter the market, with established kinase inhibitors continuing to dominate the field.

### Multi-Drug Multi-target Inhibitors (MDIs)

**Figure 2:**
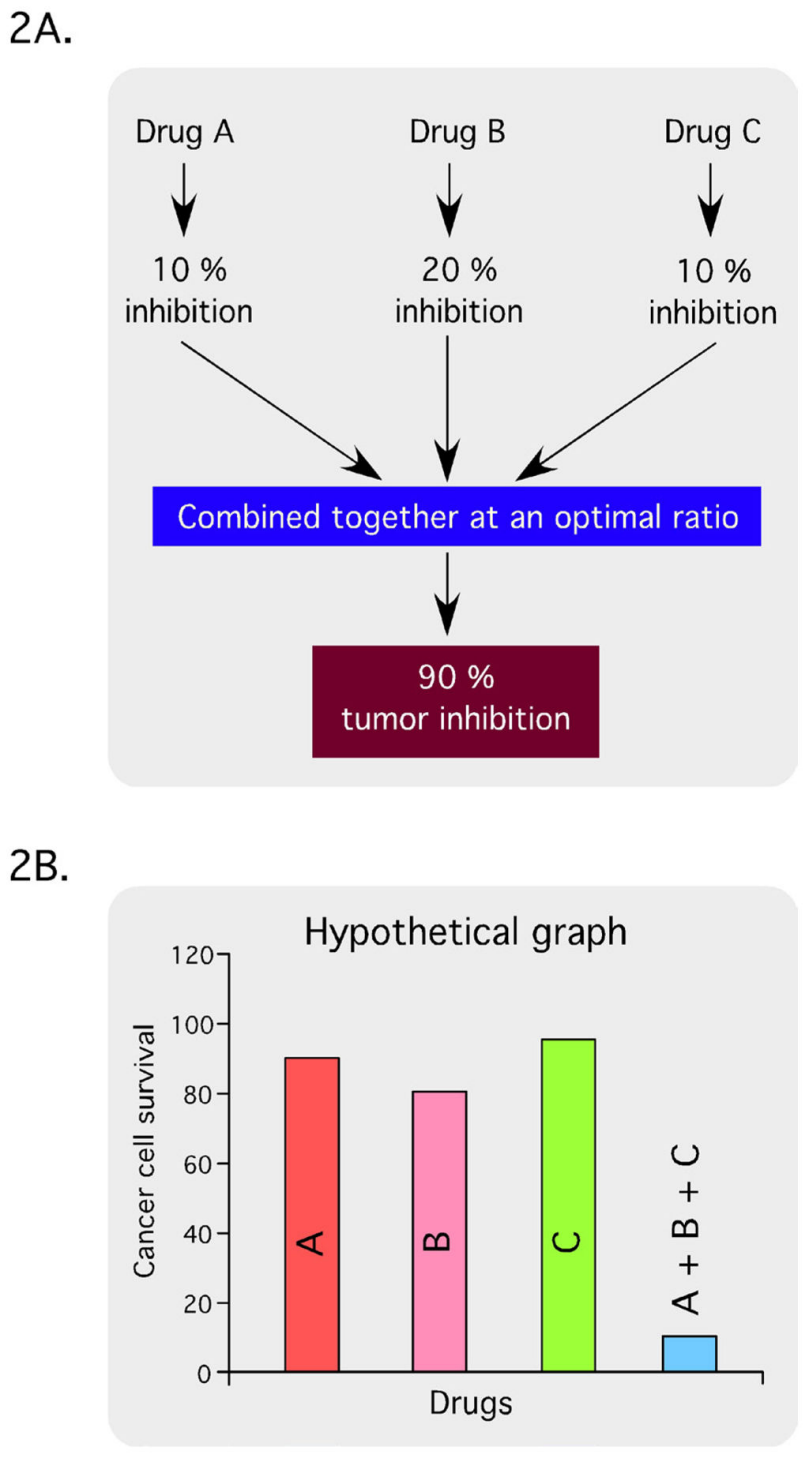
Schematic of hypothetical tumor inhibition by synergistically acting drug combinations targeting multiple key pathways.

**Figure 3:**
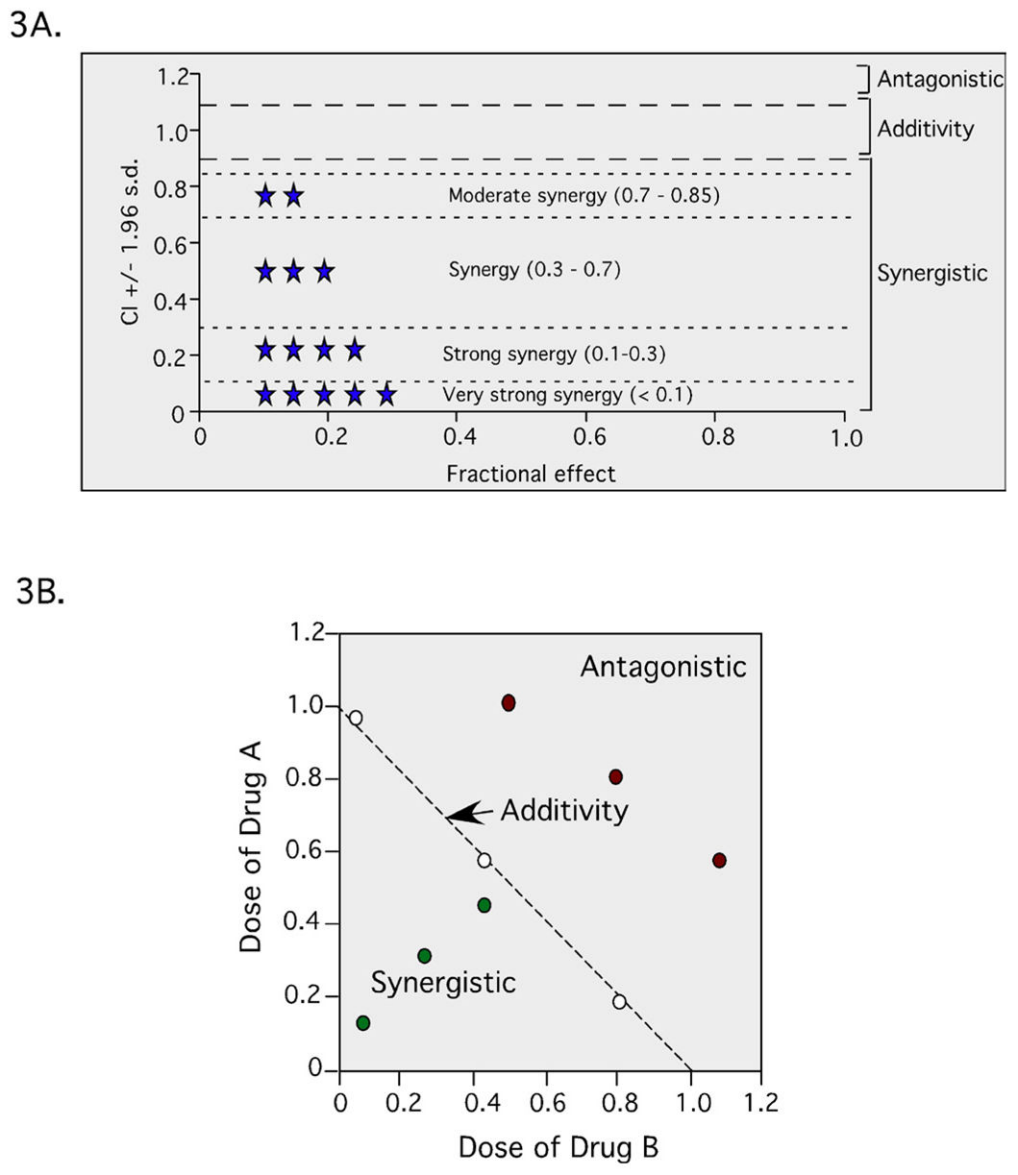
Schematic of Chou-Talalay method to determine the combination index. (3A) Combination index values of <0.85 are synergistic, 0.9–1.1 are nearly additive and >1.1 are antagonistic, and (3B) a representative isobologram showing hypothetical results that could be interpreted as antagonistic, additive or synergistic.

In addition to single-agent MTIs, synergistic drug combinations are also becoming increasingly important in cancer therapy. The hypothesis is that by combining multiple agents, a ratio for optimal inhibition of multiple targets can readily be achieved. Discovery of synergistic drug combinations can be accomplished through the use of drug synergy screens (Figure 2). In the example shown, agent A leads to 10% inhibition, agent B to 20% inhibition and agent C to 10% inhibition. However, in this hypothetical scenario, combining them causes 90% tumor inhibition. At a minimum, compound libraries for drug synergy screens should contain all active pharmaceutical ingredients (APIs) from the United States, Europe, and Japan. Over-the-counter (OTC) drugs, and Generally Recognized As Safe (GRAS) drugs and additives, as these compounds are already approved and can be moved through preclinical and clinical testing more quickly than new chemical entities [35]. When conducting these types of screens, synergy can be demonstrated using the Chou-Talalay method to determine the combination index (CI) using Calcusyn software [36,37]. Combination index values of <0.85 are synergistic, 0.9–1.1 are nearly additive, and >1.1 are antagonistic (Figure 3A), which are illustrated graphically with an isobologram (Figure 3B).

### Current clinical and preclinical development of MTIs

#### Single drug MTIs (SDIs)

There are a number of drugs currently in the market or in development that are single-agent MTIs. The various types of single-agent MTIs, the companies that market them, protein targets, and the type of cancer treated are shown (Table 1). Sorafenib is a single-agent MTI developed by Onyx and Bayer’s that targets VEGFR, PDGFR, c-Kit, and rapidly accelerated fibrosarcoma (Raf) for the treatment of RCC and HCC [38,39]. It has been shown to improve progression-free survival (PFS) and overall survival (OS) for both indications [40,41]. Novarti’s nilotinib inhibits Bcr-Abl, PDGFR, cSrc, and cKit kinases, and it has been shown to be effective in treating imatinib-resistant chronic CML [42]. Sunitinib is a multi-target tyrosine kinase inhibitor with antitumor activity has been identified as a potent inhibitor of PDGFR, VEGFR, RET, and FMS-like tyrosine kinase 3 (FLT3), and it is being used for imatinib-resistant GIST as well as advanced RCC [43]. Furthermore, CUDC-101 is a novel, small-molecule inhibitor, which simultaneously targets histone deacetylases (HDACs), EGFR, and HER2 in cancer cells. The multi-functional activity of CUDC-101 has the potential to be able to overcome drug resistance and is also currently in phase I clinical development in patients with solid tumors. Crizotinib also inhibits hepatocyte growth factor receptor (HGFR), which is an oncogene implicated in numerous cancers [44,45]. Thus, crizotinib is currently approved for NSCLC, but it is being tested in clinical trials against anaplastic large cell lymphoma, neuroblastoma, and several solid tumors in both adults and children [44,45].

Amgen & Takeda’s motesanib is being evaluated in phase II clinical trials as a first-line therapy against breast cancer. Motesanib is an inhibitor of PDGFR, VEGFR, and cKit, and earlier phase III studies against NSCLC failed to show a significant treatment-related benefit [46]. AstraZeneca’s vandetanib is a selective kinase inhibitor that selectively targets pathways critical for tumor growth and angiogenesis and being used to treat medullary thyroid cancer [30], and also currently in clinical trials with the combination of docetaxel for the treatment of NSCLC [47]. Cephalon’s lestaurtinib is in phase III trials for treatment of acute myeloid leukemia and is an inhibitor of janus kinase 2 (JAK2), FLT3, and tropomyosin receptor kinase (Trk) family kinases [48,49]. Exelixis developed cabozatinib for the treatment of medullary thyroid cancer [50], which inhibits VEGFR, cKit, FLT3, RET, and TEK. It is currently in clinical trials against numerous types of solid tumors [51,52].

GlaxoSmithKline (GSK) has developed pazopanib, a VEGFR, PDGFR, and cKit inhibitor, for the treatment of RCC and soft tissue sarcoma, and it may also be effective against NSCLC and ovarian cancer [53]. E7080 is an orally active multi-targeted kinase inhibitor whose targets include VEGFR, EGFR and PDGFR and was tested on-six human cell lines representing a number of different tumor types. However, E7080 had little effect on tumor cell proliferation but inhibits tumor angiogenesis by targeting endothelial cells. Because all of the aforementioned treatments target many pathways simultaneously, there are often severe systemic side effects when they are used. In addition, actual drug concentrations at the tumor site may be sub-optimal in many cases. With the use of a nanoparticle delivery platform, these agents may have the potential to become more effective while producing fewer side effects.

#### Multidrug MTIs (MDIs)

Combinatorial therapeutic regiments for cancer therapy have been well documented [54]. Administration of a combined array of therapeutic agents affecting different targets and displaying different toxicity profiles can lead to an improved therapeutic index [55]. While the combination therapy might be more expensive than mono-therapies, its benefits may include substantially reduced treatment failure, decreased mortality and as lower rate of drug resistance development [56]. In addition, combination therapy is also increasingly gaining in importance because it has numerous merits over conventional therapy. These merits include synergistic anticancer effects, reduced individual drug-related toxicity and reversal of drug resistance [57,58].

There are also a number of combination-MTIs on the market or in preclinical and clinical development. In some cases, combination MTIs can act synergistically to kill the cells. Recently, the mammalian target of rapamycin (mTOR) inhibitor everolimus was shown to act synergistically with gemcitabine or paclitaxel for treatment against non-Hodgkin lymphoma (NHL) cell lines [59]. The knockdown of mTOR signaling was shown to enhance the apoptosis-inducing effects of gemcitabine and paclitaxel [59]. In another study, elevated concentrations of paclitaxel were detected in brains of mice when co-administered the P-glycoprotein (P-gp) inhibitors elacridar and tariquidar [60]. Similarly, in a phase I trial, zosquidar co-delivered with daunorubicin and cytarabine increased the anticancer activity of these agents by themselves through inhibition of P-gp expression and activity [61]. Tanabe et al. [62] showed that co-delivery of mitomycin C and methotrexate significantly improved the antitumor effects against breast cancer patients pretreated with taxanes and anthracyclines. Combinations of monoclonal antibodies directed against several tumor-specific oncogenes is another method of treatment that relies on the unique ability of collections of antibodies to target cancer cells and utilize the body’s immune response to kill them through the complement cascade [57]. Expanded uses of examples of combinatorial chemotherapeutic agents are shown in Table 2.

## Impediments in the Development of MTIs Overcome through Nanotechnology

The development of MTIs for the treatment of cancer faces numerous hurdles. By using MTIs, the pharmacokinetics of the individual drugs can be coordinated and controlled, leading to optimized therapeutic activity over conventional combination treatments, which presents significant advantages for enhanced cancer chemotherapy [63]. Both types of MTIs (SDIs and MDIs) have advantages and disadvantages. For example, when using single agent MTIs, there is a customary drug development process, a standard new chemical entity intellectual property (IP) position, and easier manufacturing for a single APIs [35]. However, it can be difficult to prevent non-selectivity while achieving good potency against all intended molecular targets. Thus, there cannot be sequenced action time or dose titration when using a single agent MTI.

**Figure 4:**
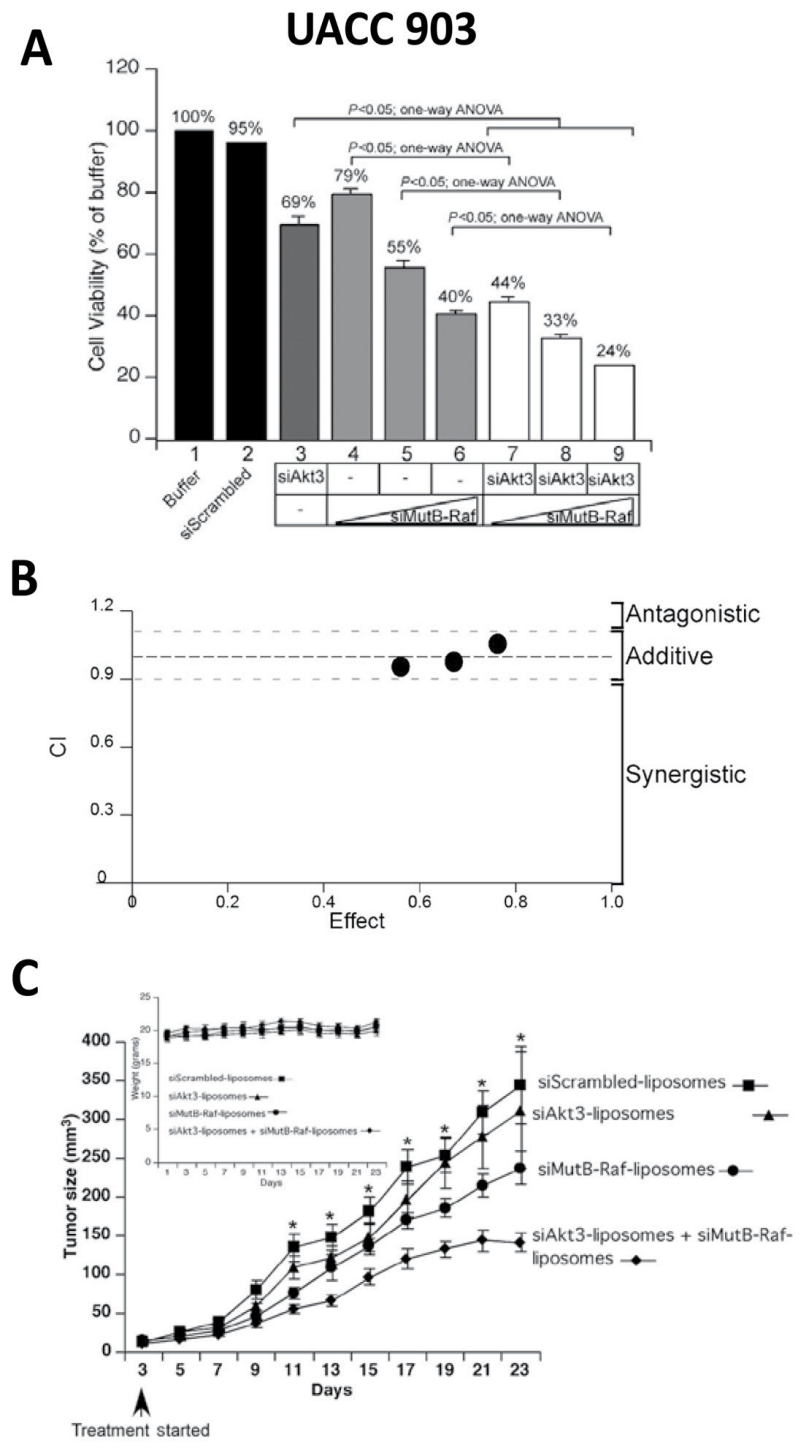
Inhibition of melanoma tumors in an additive manner following treatment with a nanoparticle containing siRNAs directed against two different targets. (4A) SiMutB-Raf and siAkt3 cooperate to reduce anchorage independent growth in cell culture. (4B) SiAkt3 and siMutB-Raf act additively to inhibit cell viability. Calculation of the CI index for the combination of siAkt3 and siMutB-Raf showed additive inhibition of cell viability with CI values between 0.94 and 1.10. (4C) Ultrasound treatment followed by topical application of siAkt3-liposomal complex containingsiMutB-Rafdecreased melanoma development in animal skin [64].

Combining multiple agents makes it much easier to tailor the ratio of the different agents to achieve optimal potency against all intended targets and also allows for sequenced action as well as varied target exposure through the use of immediate or extended release formulations [35]. For example, studies have reported that the phosphatidylinositol-3-kinase and protein kinase B (PI3K/Akt) and MAPK signaling cascades can be additively inhibited in melanoma xenograft using to small interfering ribonucleic acid (siRNA) Akt3 and V600EB-Raf loaded into cationic nanoliposomes (Figure 4). Nanoliposomes were applied topically to tumor-bearing mice that had been pretreated with low-frequency ultrasound using a lightweight four-cymbal transducer array enabling penetration of the nanoliposomal-siRNA complex throughout the epidermal and dermal layers of laboratory-generated or animal skin [64,65]. Nanoliposomal-mediated siRNA targeting of V600EB-Raf and Akt3 resulted in improved synergistic reduction in tumor size in early or invasive cutaneous melanoma compared with inhibition of each target separately with negligible associated systemic toxicity (Figure 4).

On the other hand, the increased risk of drug-drug interactions when multiple drugs are delivered simultaneously presents a serious challenge to the development of viable MDIs. By modulating mass ratios to an optimal level, these interactions can be effectively minimized while maximizing the therapeutic efficacy of the combined agents [66]. For example, poly (lactide-co-glycolide) microsphere formulations that co-deliver an antisense oligonucleotide and 5-fluorouracil (5-Fu) do not work due to the interaction of 5-FU with the oligodeoxynucleotide [66]. Each individual drug has its own distinct pharmacokinetic profile and the synergistic drug ratio for the “drug cocktails” needs to be controlled and optimized in the laboratory to reduce changes in drug dynamics, and these studies must then be replicated in animal models prior to evaluation in humans [58].

## Use of Nanotechnology to Develop MTIs

Both types of MTIs (SDIs & MDIs) have particular sets of strengths and weaknesses, but a common problem is how to achieve successful delivery and accumulation at the tumor site. Current combination therapies are limited because different drug molecules have different pharmacokinetics, biodistribution, and membrane transport properties, which creates complications in dosing and scheduling optimization [67–70]. These challenges have driven clinicians and scientists to investigate various methods of delivering multiple therapeutic agents within a single nanoparticle [71,72]. The goal of nanoparticle-based therapy is to achieve better specificity and optimized pharmacokinetics while delivering multiple therapeutic agents [73,74].

Nanoparticle-based drugs have unique properties such as small size (1–100 nm) and large surface-to-volume ratios [75]. They also benefit from self-assembly, better solubility, increased stability, and natural accumulation in the leaky tumor vasculature [73], all of which can help improve the utility of MTIs. Resistance to initially effective agents can develop because of increased metabolism, mutation of drug targets, circumvention of target pathways, or overexpression of efflux pumps [76]. By creating a nanoparticle with drug(s) that target multiple pathways, the likelihood of developing resistance decreases [77,78].

### Types of nanoparticles that could be used as MTIs

**Figure 5:**
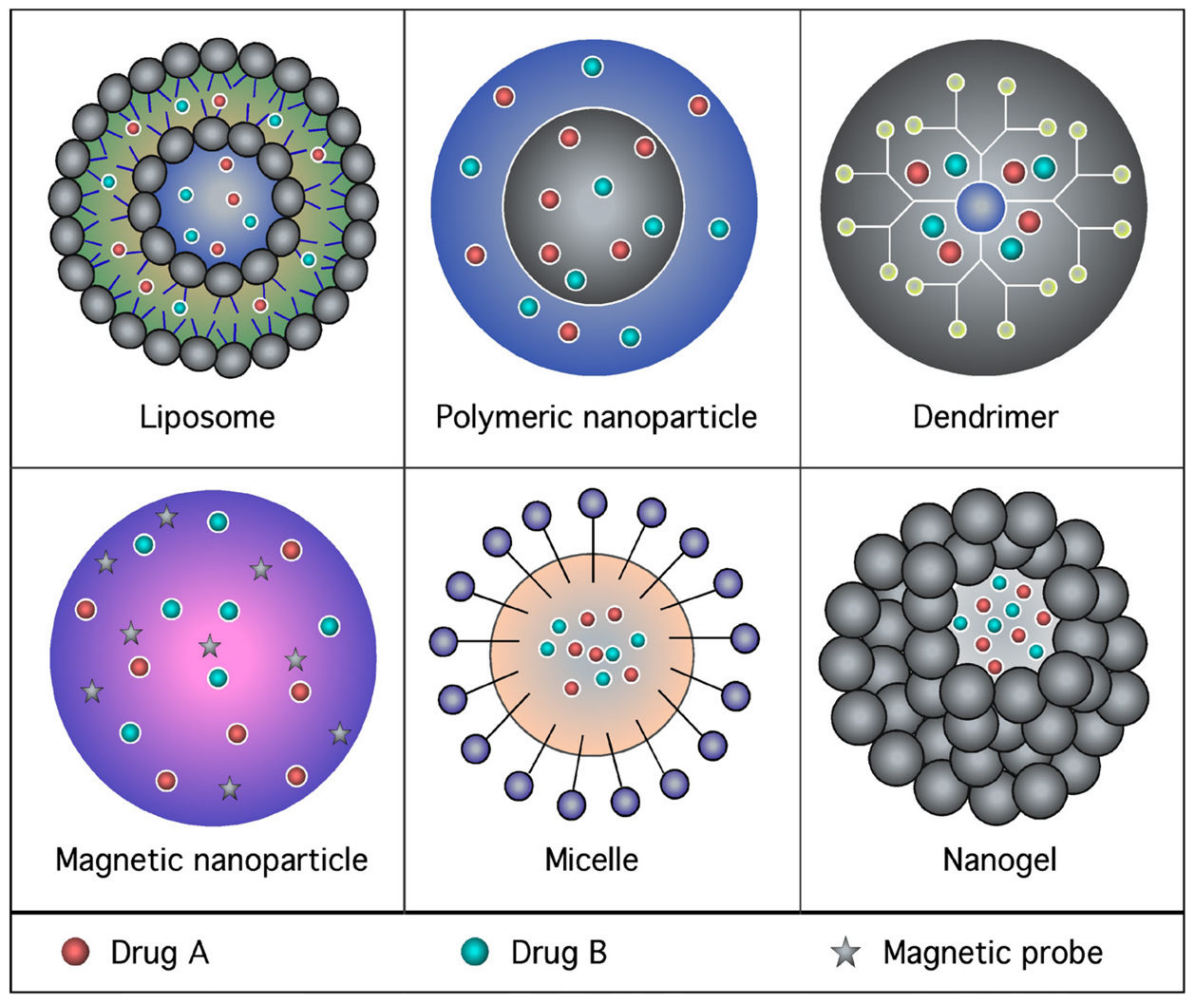
Schematic diagram representing the various types of nanoparticle use to develop MTIs. (5A) Liposome; (5B) Polymeric nanoparticle; (5C) Dendrimer; (5D) Magnetic nanoparticle; (5E) Micelle; (5F) Nanogel.

Nanoparticles are being used to circumvent many of the limitations of conventional drug delivery systems used to load single or multiple active ingredients [79–81]. The advantages include (a) delivery systems that can extend drug circulation half-life, (b) increased drug concentration at the tumor site through the passive enhanced permeation and retention (EPR) effect, (c) the ability to modify the ratio-metric dosing based on pharmacological dispositions, and (d) reduced nonspecific uptake [79,82–84]. Therefore, using nanotechnology may allow for a single platform in which multiple genetic or pharmacological agents can be loaded into nanoparticles and synergistically inhibit cancer development as well as overcome the occurrence of resistance [85–89]. Many types of nanotechnology-based therapies have been developed for treating cancers, including nanoliposomes, polymeric nanoparticles, dendrimers, magnetic nanoparticles, micelle, and nanogels (shown in Figure 5) [90–96]. These nanocarriers have been demonstrated to be capable of carrying two or more types of therapeutic payloads while promoting synergy through controlled combinatorial drug delivery. Each platform has its unique strengths and characteristics, which will be discussed briefly [97,98].

### Nanoliposomes

Nanoliposomes (shown in Figure 5) are an extensively studied drug delivery platform that is currently used in clinical practice, and it has shown promise for improving the solubility of many amphiphilic drugs [92,97,99–101]. Liposomes of certain sizes, typically less than 100–200 nm, can rapidly enter tumor sites from the blood due to EPR effect, but are kept in the bloodstream by the endothelial wall in healthy tissue vasculature [65,102–104]. Furthermore, liposomes can have various molecules attached to the surface. The most common surface modification is PEGylation to make the particle stealthy, in which polyethylene glycol (PEG) is covalently linked to the surface of the liposome [65,91,95,105,106].

PEGylated liposomes are highly stable and lead to improvement in circulation time, enhanced tumor uptake, avoidance of the reticulo-endothelial system, and minimization of toxicity [107–109]. For example, PEGylated-liposomal doxorubicin (Doxil) was characterized by a very long circulating half-life, favorable pharmacokinetic behavior and specific accumulation in tumor tissues compared with conventional liposomal doxorubicin or free doxorubicin [110,111]. Numerous liposomal drug formulations containing chemotherapeutic agents, antisense-oligodeoxynucleotides, siRNA, deoxyribonucleic acid (DNA), or radioactive particles that can target multiple signaling pathways are in various stages of development [71,92,112]. Several examples of combination drug delivery systems based on liposomes are listed in Table 3.

#### Liposomes containing nucleic acids

Nucleic acid-based nanoliposomes are used when pharmacological agents are not available to target particular oncogenic proteins. There are a large number of mutations and perturbations in cancer cells, but few play a role in disease progression. While there has been some success in designing inhibitors of specific targets, such as the use of vemurafenib against mutant ^V600E^B-Raf protein in melanoma cells [5], few targets are able to be inhibited with current technologies [113], which means that few first in class inhibitors enter the market [114]. Thus, there is great promise for siRNA-based nanoliposomal drug delivery to target these proteins, as this technique can make almost any oncogene a potential therapeutic target.

RNA interference (RNAi) blocks translation of messenger RNAs (mRNAs), thereby reducing oncogene or mutant gene protein levels. Combinations of different siRNAs targeting multiple oncogenes in different pathways may be on the horizon. For drugs such as these, it is an absolute necessity to use a nanotechnology-based method of delivery. Initial studies using localized delivery of RNAi into tumors involved viral vectors to express the siRNAs; however, limitations included several side effects, high production costs, and poor biodistribution. Nanoliposomal delivery of non-viral, less toxic methods of siRNA is being refined [115]. 1,2-Dioleoyl-3-trimethylammonium-propane (DOTAP) is one of the first cationic lipids that was created for use in liposomes for *in vivo* delivery of siRNAs [116]. These particles have a size of 60–100 nm are PEGylated (mPEG2000-C-DMA), contain cholesterol, a neutral helper lipid, and the ionizable lipid dimethylaminopropane (DLinDMA), which facilitates membrane fusion and is essential for *in vivo* efficacy of RNAi-based therapeutics [117]. Newer forms of DOTAP with 1,2-dipalmitoyl-sn-glycero-3-phosphoethanolamine-N-[methoxypoly(ethylene glycol)2000] carboxamide (DPPE-PEG2000) and egg phosphatidylcholine (egg-PC) have demonstrated serum bioavailabity for up to 20 hours [118]. Dioleoylphosphatidylcholine (DOPC)-based nanoliposomes are neutral liposomal formulations for siRNA delivery that are used against a variety of targets [119–121].

#### Liposomes combining nucleic acids and traditional pharmacological agents

Co-delivery of siRNA and chemotherapeutic agents is also another emerging area of nanoliposomal-based combination therapy [122]. For example, a positively charged cationic liposome containing siRNA in combination with doxorubicin effectively inhibits the activity of B cell lymphoma-1 (BCL-1) and multidrug-resistance-associated protein-1 (MRP1) in H69AR lung cancer lines [123]. In addition, combining nanoliposomes containing ceramide (a lipid based Akt inhibitor) with sorafenib has been shown to synergistically decrease melanoma cell growth [124]. Further studies on cancer genomes, at both the tumor and individual cell level, will enable the identification of a complete list of targets and cancer-relevant genes. By combining in-depth analysis of cancer genomes (e.g. the Cancer Genome Atlas) with RNAi technologies, there should be ample room for the growth of siRNA-based nanoliposomal therapeutic agents [125].

A recent report has described the use of trilysinoyl oleylamide (TLO)-based cationic liposomes which effectively co-delivers siMcl-1 and chemotherapeutic drug suberoylanilide hydroxamic acid (SAHA) [126]. In addition, N′,N″-dioleylglutamide-based cationic liposomes (DGL) with mitogen-activated protein/extracellular signal-regulated kinase (MEK) inhibitor PD0325901 encapsulated in lipid layers and siMcl-1 complexed to the DGL [127] has been explored. Combination treatment of PEGylated siBcl-2-lipoplex and S-1(5-FU) pro-drug has been found to exhibit enhanced antineoplastic activity in a human colorectal adenocarcinoma xenograft model [92]. Furthermore, novel fibroblast growth factor receptor (FGFR)-mediated cationic liposomes for co-delivery of doxorubicin and Msurvivin T34A plasmid have been assessed for enhanced cancer chemotherapy [128]. A recent vaccine-based approach with important implications for cancer therapy has been reported in which a liposomal delivery system carries a self-tumoral epitope (HER-2/neu-derived peptide) and CpG oligodeoxynucleotides (CpG ODN) as an adjuvant, which elicits a CD8+ mediated immune response and enhances efficacy [129].

#### Liposomes containing traditional pharmacological agents

Several nanoliposomes have been created that contain pharmacological agents and other types of compounds. Nanoliposomes containing ceramide and sorafenib have been shown to synergistically decrease melanoma cell growth [124]. Combinatorial approaches aimed at achieving greater synergistic anti-angiogenic effects have been reported by Kim et al. [130], wherein a cationic nanolipoplex has been designed to co-deliver heparin-taurocholate conjugate and SAHA. A novel polymer-lipid hybrid nanoparticle (PLN) formulation has been developed with doxorubicin and the P-gp inhibitor GG918, which can help overcome multidrug-resistant (MDR) breast cancer lines at significantly lower doses than free drugs [131]. Similarly, doxorubicin-mitomycin C co-loaded PLNs were effective in killing MDR breast cancer lines at 20–30-fold lower doses, thus indicating the potential to enhance chemotherapy and reduce the therapeutic limitations of systemic toxicity [132]. Another study Basu et al. revealed that, novel hexadentate-polyD,L-lactic acid-co-glycolic acid polymer chemically conjugated to PD98059 (MEK1 inhibitor) can significantly retard tumor development in xenograft models [133]. Dual doxorubicin-and verapamil-loaded liposomes with surface-conjugated transferrin successfully inhibited the doxorubicin-resistant K562 leukemia tumor cell line with about 5-fold greater potency compared to non-targeted, doxorubicin/verapamil loaded liposomes [134]. Since systemic injection of verapamil can cause serious cardiotoxicity, liposomal delivery of verapamil together with doxorubicin presents a promising approach to reversing cancer drug resistance and minimizing verapamil-related side effects [134]. Furthermore, alginate/bis(2-ethylhexyl) sulfosuccinate (AOT)-alginate nanoparticle-mediated photodynamic therapy using doxorubicin and methylene blue was also able to overcome resistance mechanisms in mammary adenocarcinoma tumor models, resulting in enhanced cytotoxicity against multiple drug resistant tumor cells [135].

In a phase II trial of weekly nab (nanoparticle albumin-bound)- paclitaxel (nab-paclitaxel) (Abraxane^®^) in combination with gemcitabine, established the activity and manageable toxicity as a first-line therapy of metastatic breast cancer patients [136]. Furthermore, these favorable results provide a rationale for testing nab-paclitaxel, gemcitabine, and an anti-angiogenic agent in future clinical trials [136]. In another study, Zucker et al. [137] developed a PEGylated liposomal formulation containing dual anticancer inhibitors topican and vincristine (LipoViTO), which displayed 91% tumor suppression compared with either liposomal formulations containing only one drug (40%) or a combination of free drugs (30 %) [138]. Furthermore, simultaneous liposomal delivery of quercetin and vincristine was shown to enhance estrogen-receptor-negative breast cancer treatment [139]. *In vitro* 3-[4,5-dimethylthiazol-2-yl]-2,5 diphenyl tetrazolium bromide (MTT) assays of this liposomal formulation showed significant synergism, with a combination index of 0.113 and a dose-reduction index value of 115 at ED50 for vincristine [139].

Recently, advances in liposome creation and drug-loading methods have produced specific control over combinatorial drug dosing in liposomes [140–143]. Mayer et al. [143] reported that liposomal drug combinations could be loaded at the required ratios by modifying the liposome synthesis and drug encapsulation process. This technology has yielded several products that are currently in clinical trials. For example, CPX-351 is a 5:1 cytarabine:daunorubicin dual drug loaded liposome that is currently under phase II clinical trial for the treatment of acute myeloid leukemia [144]. In addition, CPX-1, a 1:1 irinotecan:floxouridine liposome is currently under investigation in a phase II trial for colorectal cancer treatment. It exhibited superior anticancer activity in various human tumor xenograft murine models compared with liposomal irinotecan or liposomal floxouridine alone [145]. These liposomal formulations may bring a paradigm shift in clinical treatment by enabling dosage optimization in combination chemotherapy.

Palmitoyl ascorbate-modified liposomes have been described as a promising nanoparticle platform for co-delivery of paclitaxel and ascorbate, which mediates oxidative stress-induced cytotoxicity [146]. In addition, a study involving co-encapsulation of combretastatin A-4 (vascular disrupting agent) and doxorubicin (anticancer agent) suggested that a combinatorial strategy focused on arginyl-glycylaspartic acid (RGD) mediated delivery of drugs may be a promising strategy for cancer treatment [147]. Another ‘mix and match’ combinatorial treatment regimen involving a multidrug carrier (MDC) containing both gemcitabine and tamoxifen for the treatment of breast cancer has also shown good therapeutic potential [148].

Sengupta et al. [149] suggested that a nanocell drug delivery platform could enable a temporal release of multiple drugs. For this type of system, the outer PEGylated lipid envelope first releases an antiangiogenic drug shutting down the tumor vasculature and trapping the inner nanoparticle inside. The inner particle is then free to release its drug(s) targeting additional pathways in the tumor microenvironment. In addition, the liposomes can be functionalized with ligands for tumor-specific receptors, such as transferrin, folate, and integrin [150,151]. Recent advancements report the targeted nanoliposomal delivery of octreotide to the somatostatin receptor in gastric cancer [152]. On the other hand, targeted nanoconjugates are not yet effective anti-cancer agents, because they do not have easily reproducible synthesis and the surface targets display a heterogeneous intra-tumoral distribution making uniform dispersal difficult. An in-depth understanding of liposomal-drug interaction with biological system will lead to the emergence of a novel class of nanoliposomal drug delivery systems with improved anticancer activity, efficacy and safety.

### Polymeric nanoparticles

Most polymeric nanoparticles (shown in Figure 5) contain a solid, polymer-filled hydrophobic core that is better suited for water-insoluble MTI payloads [153–156]. These nanoparticles generally have higher stability, subcellular size distribution, controlled/sustained drug-release profiles, and higher loading capacity for poorly water-soluble drugs [157–159]. Polymeric nanoparticles are fabricated from biodegradable natural or synthetic polymers [98]. Several synthetic polymers are approved by the United States Food and drug administration such as poly lactic co glycolic acid (PLGA), and polycaprolactone (PCL). Other natural polymers such as chitosan, polysaccharides, and polypeptides have been investigated extensively for drug delivery and clinical applications, including cardiovascular disease, cancer, vaccines and tissue engineering [160,161]. Furthermore, these polymeric micelle systems can also be used to or concurrently deliver two or more therapeutic MTI modalities such as radiation sensitizers and drugs [79,162].

Milane et al. [163] demonstrated that epidermal growth factor receptor (EGFR)-targeted polymeric nanoparticles loaded with ionidamine and paclitaxel displayed antitumor activity through down regulation of MDR proteins in human breast and ovarian tumor cells. Furthermore, PLGA nanoparticles loaded with vincristine and verapamil triggered MDR reversal activity on MCF-7/ADR cells resistant to vincristine [164]. In another study, Misra and Sahoo [165] demonstrated that the synergistic effect of doxorubicin/curcumin PLGA nanoparticles enhances the cytotoxicity of the drugs in leukemic K562 cells *in vitro* by overcoming the MDR phenotype.

### Dendrimers

Dendrimers (shown in Figure 5) have emerged as another class of drug delivery nanoparticle platform because of their unique properties [166–168]. While these nanoparticles have not received as much attention as liposomes and polymeric nanoparticles for delivery of MTI’s, several efforts have been made to deliver multiple therapeutic agents simultaneously using a dendrimeric platform [92]. Dendrimers are globular, highly branched and synthetic polymers that are characterized by a central inner core surrounded by repetitive layers and an outermost layer of multivalent functional groups [166,167]. The high level of control over the architecture affecting size, shape, density, and surface functionality makes these compounds excellent carriers of MTIs as well as imaging agents through chemical modification of multiple terminal groups [169–172].

Despite the promise of dendrimers, a major constraints for delivery of MTIs is toxicity due to the interaction of surface cationic charges with negatively charged biological membranes *in vivo*, which can cause membrane nanoholes [173]. Surface engineering can be used to mask the cationic charges, which involves PEGylation, acetylation, and carbohydrate or peptide conjugation. The chemical modifications necessary to overcome the toxicity have been discussed by others [173,174]. The synthesis of dendrimers can be tightly controlled to establish specific size range and branching complexity. Drug could be loaded into the core or branches [175]. The dendritic surface can be further modified with antibodies or ligands to improve targeting and utility of these nanoparticles to carry anti-cancer agents [176].

The unique properties of dendrimers make them a desirable platform for simultaneous delivery of hydrophobic and hydrophilic MTI’s [171,172]. Tekade et al. [177] developed dual drug-loaded polyamidoamine dendrimers loaded with hydrophobic methotrexate and hydrophilic all-trans retinoic acid. These particles exhibited less hemolytic toxicity and enhanced cytotoxicity in HeLa cells compared to free drugs. In another study, Kaneshiro et al. formulated novel nanoglobular dendrimers conjugated with cyclo(-Arg-Gly-Asp-d-Phe-Lys-(cRGDfK) peptide with PEG spacer for co-delivery of doxorubicin (DOX) and siRNA. The siRNA complex of the targeted conjugates resulted in higher gene silencing efficiency in glioblastoma U87-Luc cells and greater efficacy than either agent alone [178]. Other examples of dendrimer-based combination cancer therapy are summarized in Table 4.

### Magnetic nanoparticles

Magnetic nanoparticles (MNPs) (shown in Figure 5) are a major class of nanoparticles with the potential to enhance magnetic resonance imaging (MRI), targeted therapy, tissue repair, virus detection, magnetically enhanced transfection, magnetically induced hyperthermia, cell/protein/DNA separation and radiotherapy [179–183]. MNPs are spherical nanocrystals of 10–100 nm in size with an Fe2+ or Fe3+ core surrounded by lipids, liposomes, proteins, polymers, or dextran and surface-coated with non-polymeric stabilizers, providing the opportunity for the smart delivery of therapeutic materials.

Iron oxide MNPs (magnetite, Fe_3_O_4_; maghemite, Fe_2_O_3_) are extensively used as the core of magnetic nanocarriers due to super paramagnetic properties and biocompatibility [184–186]. Iron oxide provides significant advantages over traditional contrast agents, including high magnetic signal strength, relatively low cytotoxicity, longer lasting contrast enhancement, and improved delineation of tumor margins as well as low sensitivity to the number of surrounding water molecules [187–189]. While copper, cobalt and nickel are also highly magnetic materials, the chemical composition makes them naturally toxic and more susceptible to oxidation. Thus, they have limited utility for the MTIs based applicaitons. In contrast, titanium and iron oxide-based particles are considered significantly less damaging to cells and could have utility of delivery of MTIs [190,191].

Currently, various studies have investigated the potential application of magnetic nano systems for pharmaceutical and biomedical applications [192–194]. Jaemoon et al. developed an anti-HER2 antibody conjugated to multifunctional magneto-polymeric nanohybrids (MMPNs) encapsulated by an amphiphilic block copolymer that showed excellent synergetic effects for inhibition of tumor growth and simultaneous breast cancer imaging [195]. Furthermore, Yu et al. [196] has developed doxorubicin-loaded thermally cross-linked superparamagnetic iron oxide nanoparticles (Dox@TCL-SPION) and demonstrated simultaneous detection of tumors by magnetic resonance imaging and delivery of anticancer drugs via release from nanoparticles. This strategy exhibited exceptional antitumor effects without any systemic toxicity. Kumar et al. [197] synthesized and characterized novel hybrid MNPs containing hyaluronic acid (HA) and iron oxide, and successfully delivered nearly 100 % in HEK293 and A549 cell lines, which is an encouraging development in effective tissue and cell targeting systems. Murali et al. developed MNPs that are loaded with curcumin as the anticancer agent and used them for simultaneous targeting and imaging in breast cancer cell lines [198]. In addition, Shi et al. [200] developed a multifunctional drug delivery system, which is based on covalently attaching genistein into iron oxide (Fe3O4) nanoparticles coated by cross-linked carboxymethylated chitosan (CMCH). Results suggest that these particles improve the inhibitory effects of SGC-7901 in cancer cells relative to the free drug.

### Micelle

A micelle composed of numerous amphiphilic surfactant molecules is emerging as a promising class of MTIs anticancer nanoparticles (shown in Figure 5) [200]. A mixture of hydrophobic interactions, electrostatic interactions, metal complexation, and hydrogen bonding of block copolymers drives the process of micellization in aqueous solutions [201]. The core of the micelle can be a storage area for drugs with many different properties. The outer core can be functionalized to improve its drug-like qualities and also to modify its physicochemical characteristics [201]. The fundamental mechanisms of self-assembly, drug loading/release, stability, and intracellular delivery of micellar formulations has been reviewed in the literature [202,203]. However, to translate micellear formulations into widespread clinical applications, a better understanding of the physicochemical properties of this type of nanoparticle is still needed [204].

Polymeric micelles are a subset of micelles comprised of block copolymers consisting of both hydrophilic and hydrophobic monomeric units [205]. The first clinical polymeric micelle formulation received approval in South Korea and is currently undergoing phase II trials in the United States where it is known as Genexol-PM64 (PEG-poly (D,L-lactide)- paclitaxel) [206,207]. A biodegradable cationic nanomicelle based on a triblock copolymer of poly (N,N-dimethylamino-2-ethyl methacrylate)-polycaprolactone-poly(N,N-dimethylamino-2-ethyl methacrylate) (PDMAEMA-PCL-PDMAEMA) was reported by Zhu et al. [208]. This nano-micellar drug delivery system has paclitaxel loaded in its micellar core while siRNA is complexed to the outer PDMAEMA shell of the micelle [208].

A recent report describes a hybrid polymeric micelle consisting of a PEG-phospholipid block copolymer with an envelope containing the anti-angiogenesis agent combretastatin surrounding an inner PLGA nanoparticle bearing the chemotherapeutic agent doxorubicin [149,209]. Furthermore, simultaneous delivery of two chemotherapeutics agents paclitaxel and 17-allylamino-17-demethoxygeldanamycin (17-AAG) was achieved by employing PEG-distearoylphosphatidylethanolamine/tocopheryl polyethylene glycol 1000 (PEG-DSPE/TPGS) mixed micelles [210]. Fan et al. [211] designed multifunctional micellar nanoparticles containing pyrrolidine dithiocarbamate (PDTC) and doxorubicin with the goal of simultaneously delivering the chemotherapeutic agents and bypassing the multidrug resistance proteins. Co-delivery of survivin small hairpin RNA (shRNA) and paclitaxel has been used by Hu et al. for its synergistic cytotoxic effects in ovarian cancer therapy [212]. Another study by Wiradharma et al. [213] reported the production of [Ac-(AF)_6_-H_5_-K_15_-NH_2_] FA32 micelles with enhanced potential to deliver the hydrophobic anticancer drug doxorubicin and the p53 gene simultaneously.

### Nanogels

Nanoscale hydrogels or nanogels are biocompatible, three-dimensional materials consisting of hydrophilic, cross-linked polymer networks that can be loaded with therapeutic agents (shown in Figure 5) [214, 215]. This unique drug delivery system is characterized by a controlled release mechanism, which is dependent on the diffusion coefficient of the drug through the hydrogel network [216]. Hydrogel nanoparticle systems are an important constituent of a new class of drug delivery system popularly known as “intelligent,” or “smart” or “stimuli-responsive” drug delivery systems. The environmental stimuli include changes in pH, temperature or ionic strength [217–219]. While the nanogel matrix bearing the drug is in a collapsed state, stimuli cause the nanogel to swell, leading to an increase in mesh size and change the drug diffusion rate [220,221].

Nanogels have many characteristics that could be associated with an ideal drug delivery platform, including stability, response to biologically relevant stimuli, passive and active targeting, low toxicity, and ease of synthesis [222]. Nanogels can be prepared from polymer precursors or by fabricating them via heterogeneous polymerization of monomers [222]. In the first method of generation, amphiphilic copolymers are allowed to self-assemble in solution, and then the assembly is ‘locked’ via some form of cross-linking [222]. Polymeric nanogels can also be easily functionalized with cell-targeting ligands, and sizes can be controlled for various drug delivery applications [222]. Parenteral delivery of nanogels is possible since they can easily be delivered in a liquid form [223]. Seo et al. [224] recently reported a chemo-immunotherapeutic strategy to target cervical cancer that employs a biodegradable chitosan-based hydrogel that can codeliver chemotherapeutic agent such as doxorubicin, cisplatin, or cyclophosphamide with an immunoadjuvant-granulocyte macrophage colony-stimulating factor (GMCSF).

## General Constraints and Challenges in MTIs Nanoparticle Development

Particle size is the most important and most studied factor affecting nanoparticle toxicity and success of MTIs. If nanoparticles are too small (e.g. <10nm), they can pass the blood brain barrier and cause damage. In contrast, particles >100nm do not possess the desired pharmacological properties for MTIs effective drug delivery [225]. The charge and composition of a MTIs nanoparticle can also have a profound impact on its toxicity. Positively charged nanoparticles can be toxic due to hemagglutination and hemolysis of erythrocytes [225]. They can also agglomerate due to intermolecular forces, to further increase toxicity. Methods have been developed to circumvent this potentially serious side effect, involving sonication, use of detergents, and PEGylation of the nanoparticle [225]. MTIs nanoparticle-related toxicities can vary based on each nanoparticle and in these cases more in depth studies to explore the structural properties that induce organ-specific cytotoxicity would be needed [225].

PEGylation to reduce toxicity, improve biodistribution and half-life of the agent can be used to mask toxic nanoparticles. Many studies have conjugated functional groups to nanoparticles in order to improve targeting, biodistribution, and reduce toxicity. In addition to toxicity, the lack of reproducibility of large-scale commercial production is another major drawback to MTIs based nanoparticles development [225].

## Conclusion

The use of MTIs for the treatment of cancer should significantly enhance cancer therapy. The key challenge in development of MTIs based nanotherapeutics lies in optimization of drug loading and controlled release of the encapsulated cargo. In order to circumvent these limitations and to optimally pair the nanocarrier and the therapeutic agent, a greater understanding of the interplay between properties of the carrier and the mechanisms of loading is key [216]. The major driving forces for the development of MTI nanotherapeutics include enhanced efficacy, improved drug delivery, and reduced development of recurrent resistant disease.

If successful, MTIs based nanotherapeutics could revolutionize the future of cancer treatment. Single agent MTIs and combinatorial strategies involving synergistically acting multidrug MTIs have numerous advantages over conventional cancer pharmaceutics. Even though different types of nanoparticles are being developed for the delivery of MTIs, nano-liposomal drug delivery platforms are currently the most developed and versatile nanotechnology based on increased clinical approval of liposomal chemotherapeutic agents.

## Figures and Tables

**Table 1:** Single-agent MTIs currently undergoing preclinical and clinical use.

Agent	Company	Indication	Targets	References
Sorafenib	Onyx/Bayer	RCC, HCC	VEGFR, PDGFR, c-Kit, Raf	[38–41]
Nilotinib	Novartis	CML	Bcr-Abl, PDGFR, cSrc, c-Kit	[42]
Sunitinib	Pfizer	GIST, RCC	PDGFR, VEGFR, c-Kit, RET, FLT3	[43]
Crizotinib	Pfizer	NSCLC	EML4/ALK, HGFR	[44, 45, 226]
Motesanib	Amgen/Takeda	Breast cancer	PDGFR, VEGFR, c-Kit	[46]
Vandetanib	Astra Zeneca	Thyroid, NSCLC	EGFR, VEGFR, RET	[30]
Lesaurtinib	Cephalon	AML	JAK2, FLT3, Trk	[48, 49]
Cabozatinib	Exelixis	Thyroid, solid tumors	VEGFR, MET, c-Kit, FLT3, RET, TEK	[51, 52]
Pazopanib	GlaxoSmithKline	RCC, sarcoma	VEGFR, PDGFR, c-Kit	[53]

**Abbreviations:** RCC: Renal Cell Carcinoma; HCC: Hepato Cellular Carcinoma; CML: Chronic Myelogenous Leukemia; GIST: Gastro-intestinal Stromal Tumor; NSCLC: Non-small-cell Lung Cancer; AML: Angiomyolipoma; VEGFR: Vascular Endothelial Growth Factor Receptor; PDGFR: Platelet-derived Growth Factor Receptor; cKit: Mast/stem Cell Growth Factor Receptor; cSrc: tyrosine-protein Kinase; Bcr-Abl: Break Cluster Region-Abelson Leukemia; RET: REarranged during Transfection; FLT3: FMS-like Tyrosine Kinase 3; EML4: Echinoderm Microtubule-associated protein-like 4; ALK: Anaplastic Lymphoma Kinase; HGFR: Hepatocyte Growth Factor Receptor; JAK2: Janus Kinase 2; Trk: Tropomyosin Receptor Kinase; TEK: Tyrosine Endothelial Kinase

**Table 2:** Combination MTIs currently undergoing preclinical and clinical trials.

Agents	Indication	Reference
Everolimus + Gemcitabine or Paclitaxel	Non-Hodgkin’s Lymphoma	[59]
Daunorubicin + Cytarabine + Zosquidar	Angiomyolipoma	[61]
Mitomycin C + Methotrexate + Taxanes	Breast cancer	[62]
5-Fluorouracil + Leucovorin	Colon cancer	[227]
Paclitaxel + Carboplatin	Non-small-cell lung cancer	[228]
Exemestane + Zoledronic acid	Breast cancer	[229]

**Table 3:** Liposomes for combination therapy.

Nanocarrier system	Agents	Indication	Status	References
Liposome (CPX-351)	Cytarabine + Daunorubicin	Advanced Hematologic Cancer	Phase II	[144]
Liposome (CPX-1)	Irinotecan + Floxuridine	Advanced Colorectal Cancer	Phase II	[145, 230]
Liposome (CPX-571)	Irinotecan and Cisplatin	Non-small-cell lung cancer	Preclinical	[231]
PEG- Liposome	Topotecan + Vincristine	Brain cancer	Preclinical	[138]
Liposome	Topotecan + Amlodipine	Leukemia	Preclinical	[232]
Liposome	Vincristine + Quinacrine	Leukemia	Preclinical	[233]
Liposome	6-Mercaptopurine + Daunorubicin	Leukemia	Preclinical	[234]
Liposome	Paclitaxel + Tariquidar	Ovarian cancer	Preclinical	[235]
Transferrin-conjugated PEGylated liposome	Doxorubicin + Verapamil	Leukemia	Preclinical	[134]
Trilysinoyl oleylamide(TLO)-based cationic liposomes	siMcl1 + Suberoylanilide hydroxamic acid	Cervical cancer	Preclinical	[126]
Nanolipoplex	Taurocholate(LHT7)+ Suberoylanilide hydroxamic acid	Oral cancer	Preclinical	[130]
Liposome	PD0325901 +siMcl1	Cervical cancer	Preclinical	[127]
Liposome	Doxorubicin+ Msurvivin T34A plasmid	Lung carcinoma	Preclinical	[128]
Liposome	Ceramide+ Sorafenib	Breast Cancer	Preclinical	[124]
Liposome	siB-Raf + siAkt3	Melanoma	Preclinical	[236]
PEGylated lipoplex	siBcl-2-lipoplex+ S-1(5-FU) pro-drug	Colorectal cancer	Preclinical	[92]
RGD-modified liposomes	Combretastatin A-4 + Doxorubicin	Melanoma	Preclinical	[147]
Liposome	Gemcitabine + Tamoxifen	Breast cancer	Preclinical	[148]

**Table 4:** Nanoparticles based combination therapy.

Nanocarrier system	Agents	Indication	Status	Reference
RGDfK-G3 Poly-lysine dendrimer	Doxorubicin + siRNA	Glioblastoma	Preclinical	[178]
Dendritic PEG	Paclitaxel + Alendronate	Cancer bone metastases	Preclinical	[237]
Folate-G5 poly-propyleneimine dendrimer with ethylenediamine core	Methotrexate + all-trans-retinoic acid	Leukemia	Preclinical	[238]
G5 PAMAM dendrimer	Antisense-miRNA21 + 5-Fluorouracil	Glioblastoma	Preclinical	[239]
Aptamer-G4 PAMAM dendrimer conjugates	Unmethylated CpG-ONTs + Doxorubicin	Prostate	Preclinical	[240]
PLGA	Vincristine + Verapamil	Hepatocellular carcinoma	Preclinical	[241]
Methoxy PEG-PLGA	Doxorubicin + Paclitaxel	Various cancer	Preclinical	[242]
PLGA-PEG-biotin	Paclitaxel + Tariquidar	Various cancer	Preclinical	[243]
PLGA-PEG-biotin	Paclitaxel + P-gp siRNA	Various cancer	Preclinical	[244]
HPMA-Gem-Dox	Gemcitabine +Doxorubicin	Prostate cancer	Preclinical	[245]
HER2 conjugated- GMO-MNPs	Paclitaxel + Rapamycin	Breast cancer	Preclinical	[246]
Ac-(AF)_6_-H5-K_15_-NH_2_ (FA32) micelle	Doxorubicin + p53 gene	Hepatocarcinoma	Preclinical	[213]

**Abbrviations:** RGD: Arginylglycylaspartic Acid; siRNA: Small Interfering Ribonucleic Acid; miRNA: Micro Ribonucleic Acid; HER2: Human Epidermal Growth Factor Receptor 2; PEG: Polyethylene Glycol; PLGA: Poly(Lactic-co-glycolic Acid); PAMAM: Polyamidoamine; HPMA: Poly(N-(2-hydroxypropyl)methacrylamide); MNP: Magnetic Nanoparticles
